# Simultaneous bile duct and portal vein ligation induces faster atrophy/hypertrophy complex than portal vein ligation: role of bile acids

**DOI:** 10.1038/srep08455

**Published:** 2015-02-13

**Authors:** Weizheng Ren, Geng Chen, Xiaofeng Wang, Aiqun Zhang, Chonghui Li, Wenping Lv, Ke Pan, Jia-hong Dong

**Affiliations:** 1 Department & Institute of Hepatobiliary Surgery, Chinese PLA General Hospital, Beijing, China; 2 Department of Hepatobiliary Surgery, Southwest Hospital, Third Military Medical University

## Abstract

Portal vein ligation (PVL) induces atrophy/hypertrophy complex (AHC). We hypothesised that simultaneous bile duct and portal vein ligation (BPL) might induce proper bile acid (BA) retention to enhance AHC by activating BA-mediated FXR signalling in the intact liver and promoting apoptosis in the ligated liver. We established rat models of 90% BPL and 90% PVL and found that BPL was well-tolerated and significantly accelerated AHC. The enhanced BA retention in the intact liver promoted hepatocyte proliferation by promoting the activation of FXR signalling, while that in the ligated liver intensified caspase3-mediated apoptosis. Decreasing the BA pools in the rats that underwent BPL could compromise these effects, whereas increasing the bile acid pools of rats that underwent PVL could induce similar effects. Second-stage resection of posterior-caudate-lobe-spearing hepatectomy was performed 5 days after BPL (B-Hx), PVL (V-Hx) or sham (S-SHx), as well as whole-caudate-lobe-spearing hepatectomy 5 days after sham (S-Hx). The B-Hx group had the most favourable survival rate (93.3%, the S-SHx group 0%, the S-Hx group 26.7%, the V-Hx group 56.7%, P < 0.01) and the most sustained regeneration. We conclude that BPL is a safe and effective method, and the acceleration of AHC was bile acid-dependent.

Partial liver resection is often the only curative option for various benign and malignant liver diseases^1^, but many patients are evaluated as unable to tolerate the procedure due to an insufficient future remnant liver (FRL). To acquire resectability, portal vein ligation (PVL) has been established as a routine procedure. After PVL, the ligated liver underwent atrophy, while the intact liver underwent hypertrophy, or atrophy/hypertrophy complex (AHC)^2^. According to previous reports, extended hepatectomy would also be better tolerated after PVL^3^. However, PVL is associated with a prolonged waiting and frequent poor responses clinically. Improved methods are needed.

Bile acids (BAs) function as mitogenic and signalling molecules for hepatocytes^4^. After liver loss, homeostasis of BAs needs to be tightly controlled to prevent cholestasis while allowing proper BA signalling to enable regeneration^5^. BAs could initiate and/or promote hepatocyte proliferation via the activation of primary nuclear BA receptor FXR, and the BA signalling is dispensable for normal liver regeneration^6^,^7^. During the early phase after PHx, the remaining hepatocytes are exposed to an increased BA load, which initiates a series of regenerative and protective responses^5^,^8^,^9^,^10^. If the BA overload was prohibited, liver regrowth would be compromised^4^. After PVL, the lobes with portal vein ligated could compensate for bile drainage. It can be postulated that the absence of BA retention might compromise regenerative capacity. If a proper amount of BA retention could be introduced, the regeneration of the intact liver might be enhanced. On the other hand, BAs are toxic and apoptotic, substantial increases in hepatic BA levels, as in cholestatic liver disease, could induce and/or aggravate necrosis and apoptosis^4^,^11^. The introduced BA retention should be moderate in the intact liver as not to cause intolerable liver damage^8^,^9^,^10^. Furthermore, BA retention in the ligated liver might also promote the apoptosis-mediated atrophy, which could, in turn, further promote regeneration of the intact liver. Thus, by introducing an appropriate elevation of BA concentration in the PVL model, the AHC might be further boosted.

Bile duct ligation is a classic model of inducing BA retention. After PVL, deprivation of the portal feeding could significantly reduce the BAs entering the ligated liver. If the bile duct of the same portion was ligated or had simultaneous bile duct and portal vein ligation (BPL), induced BA retention should be tolerable. We hypothesise that BPL could induce stronger AHC via activating BA-mediated FXR signalling in the intact liver and promoting apoptosis in the ligated liver. To test our hypothesis, we established rat models of BPL and PVL and compared their effects in inducing AHC and the tolerance of a hypertrophied liver with second-stage extended hepatectomy. Furthermore, the role of BAs in enhancing AHC was examined more directly in rats in which BA pools were either increased or decreased when they received PVL/BPL.

## Methods

### Animal models

**Figure 1:**
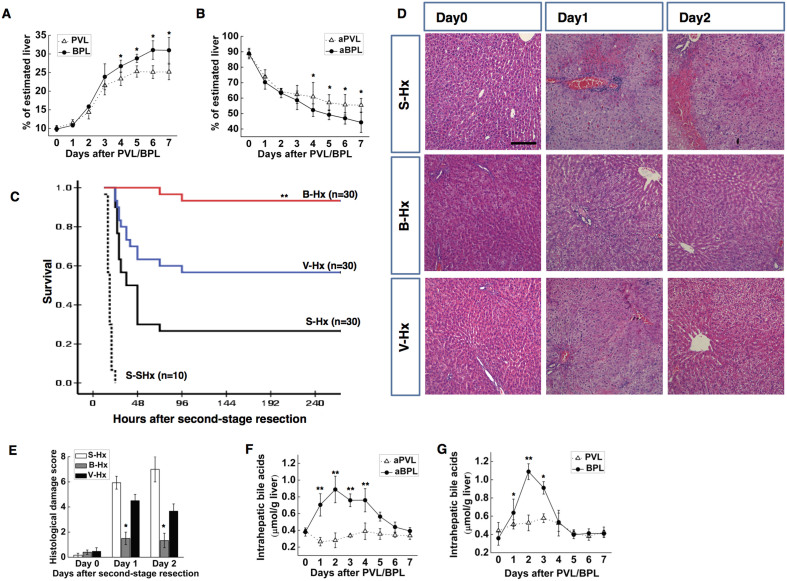
BPL induced faster AHC than PVL. Rats underwent BPL before extended hepatectomy showed the most favourable prognosis. Hepatic bile acid levels were distinctive. Time profile of the change in the weight of the hypertrophied liver (**A**) and the atrophied liver (**B**) After BPL/PVL. Day 5 was chosen as the time for second-stage hepatectomy. Survival after staged hepatectomy (**C**). Representative images of H&E-stained liver sections after hepatectomy (**D**). Samples were scored for the histological damages (vacuolation and necrosis) as described (**E**). The damage was most prominent in S-Hx group, and they were alleviated in the other groups, particularly B-Hx. Changes of hepatic bile acids in the hypertrophied liver after BPL/PVL (**F**), and in the liver remnant after hepatectomy (**G**) (Scale bar, 100 μm. The data are presented as the mean ± SD, *p < 0.05, *p < 0.01).

The experiments were performed on 7-week-old male Sprague-Dawley (SD) rats, weighing 220–250 g (purchased from the Laboratory Animal Research Center of the Academy of Military Medical Science). This study and all experimental protocols were approved and the methods were carried out in accordance with the guidelines of the Institutional Animal Care and Use Committee of the Chinese PLA General Hospital. The procedures were performed as illustrated (Si-Fig. 1A), with details described in *SI-Materials and Methods*. Rats underwent Sham/PVL/BPL to investigate the atrophy and hypertrophy of the liver, and second-stage hepatectomy preserving the posterior caudate lobe was performed on day 5 post-sham (Sham subtotal hepatectomy; S-Hx), PVL (PVL-Hepatectomy, V-Hx) and BPL (BPL-Hepatectomy, B-Hx). The S-Hx group (Sham-hepatectomy, S-Hx) underwent the sham procedure followed by whole-caudate-lobe-sparing hepatectomy. Rats were sacrificed as indicated (SI-Fig. 1B). In a separate study, 30 rats were used to examine the survival rate in each of the four groups. To investigate whether BAs were responsible for the effect induced by BPL, diets containing taurocholate (0.2%, Sigma-Aldrich, St. Louis, Missouri) or cholestyramine (2%, Sigma-Aldrich) were fed to rats that underwent PVL or BPL (SI-Fig. 1C).

### Liver weight, serum laboratory tests and hepatic bile acids

The weights of the rats were measured before sacrifice. Liver weight was calculated into the percentage of estimated total liver weight. Hepatic bile acids were extracted as described^4^. The hepatic bile acid levels and serum markers were measured using a serum analyser (Cobas-Mira Plus; Roche, Basel, Switzerland).

### Histology and Immuno-staining

After HE staining, the samples were scored for liver histological damages (necrosis + vacuolisation) in 20 random visual fields (400x), in accordance with Suzuki's criteria^12^. The scoring systems were: necrosis 0, none; 1, single cell necrosis; 2, <30%; 3, 30% ~ 60%, 4, >60%; vacuolisation, 0, none; 1, minimal; 2, mild; 3, moderate; 4, severe. For immunofluorescence analysis, slides were incubated with antibodies including rabbit anti-cleaved caspase-3 (1/3000, Cell Signaling Technology, Boston, MA), mouse anti-Ki-67 (1/50, BD Pharmingen, San Diego, CA) rabbit anti-CK19 (1/200, Sigma-Aldrich). After washing with PBS, the slides were incubated with secondary antibodies for detection of rabbit antibodies (1:500; Alexa-594 or Alexa-488; Invitrogen, California, Carlsbad). Nuclei were visualised with DAPI (40,6-diamidino-2-phenylindole, Sigma-Aldrich). Confocal images were acquired using an Olympus Fluoview FV1000 (Tokyo, Japan). The number of cleaved caspase3-stained hepatocytes was determined in 20 random visual fields (200x). The percentage of stained/total hepatocyte nuclei was designated as the cleaved-caspase-3 labelling index. For Ki-67 staining, slides were stained with mouse anti-human Ki-67 (1/50, BD Pharmingen, San Diego, CA), and the Polink-2 plus Polymer HRP detection system was used (Golden Bridge International, Mukilteo, WA). The number of Ki-67 positive hepatocytes was determined in 20 random visual fields (400x). The percentage of stained/total hepatocyte nuclei was designated as the Ki-67 labelling index.

### Western blotting analysis

Following SDS/PAGE, transfer and blocking, the PVDF blots were incubated with primary antibodies, including rabbit anti-caspase-3, anti-P21 antibody (1/500 Santa Cruz Biotechnology, Santa Cruz, CA), mouse anti-Farnesoid X Receptor (1/1000 Invitrogen) and mouse anti-GAPDH antibody (1/5000, Sangene Biotech Co, Tianjin, China). The membranes were washed and incubated with a secondary antibody from Santa Cruz, developed with a Super-Signal chemiluminescent substrate. Densitometry was measured using the Image J program (National Institutes of Health, Bethesda, MD).

### Quantitative Real-Time Polymerase Chain Reaction

The total RNA was extracted from the frozen liver tissues using RNASimple Total RNA Kit (Tiangen Biotech, Beijing, China) according to the manufacturer's protocol. A total of 4 μg of RNA was reverse-transcribed with the RevertAid First Strand cDNA Synthesis Kit using Oligo-dT primers (Thermo Scientific, Waltham, MA). Quantitative real-time polymerase chain reaction (qPCR) amplifications were performed with the SYBR Premix Ex Taq II (TakaRa, Tokyo, Japan) on the StepOnePlus Real Time PCR System (Applied Biosystems, Foster City, CA). The primers are shown in Table 1. The relative mRNA expression levels were normalised to the endogenous reference gene glyceraldehyde-3-phosphate dehydrogenase (GAPDH) and calculated using the 2-ΔΔCt method as previously described^13^. The mRNA levels of the experimental groups were expressed as fold changes versus those underwent the sham operation.

### Statistical analysis

All data are presented as means ± SD. The statistical analysis was performed using SPSS statistical software, version 17.0 (SPSS Inc, Chicago, IL). Differences between means were assessed using Student's t test, Kaplan-Meier method, rank-sum test or by one-way ANOVA when required. Differences were considered significant at p < 0.05.

## Results

### BPL induced faster atrophy/hypertrophy complex(AHC) than PVL, and significantly improved the safety of extended second-stage hepatectomy

We established rat models of 90% BPL and 90% PVL (SI-Fig. 1A). All the rats survived. Compared with their initial weights, there was a slight but insignificant weight loss in both groups, which was compensated within the observation period. There was no evident bile duct dilation in any of the rats.

**Figure 2:**
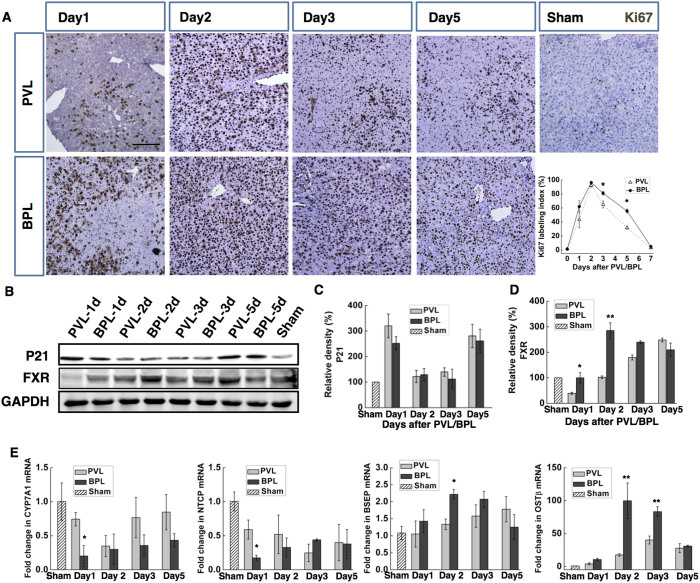
BPL induced well-tolerated promotion of proliferation in the intact liver than PVL, involving FXR-dependent bile acid signalling. Ki67 immunohistochemical staining of the hypertrophied portion after BPL/PVL, with the Ki-67 labelling indexes (**D**).Western blotting for p21 expression of the hypertrophied lobes after PVL/BPL, revealed no significant difference between the groups(**B,C**). Western blotting for FXR revealed earlier activation in the BPL group(B,D) Densitometric measurements were acquired from 3 individual experiments.CYP7a1, NTCP, BSEP and OSTβ mRNA expression levels were measured in the intact liver by quantitative real-time PCR (**E**). (Scale bar, 100 μm. The data are presented as the mean ± SD, *p < 0.05, **p < 0.01, Full-length blots are presented in SI-Figure 4).

BPL induced a faster and more sustained AHC than PVL (Fig. 1A,B). After day 4 post-ligation, the difference in the weight of the intact and ligated liver became significant between the two groups. Compared to PVL, BPL led to a transient aggravation of the serum liver damage markers, alanine aminotransferase (ALT) and aspartate aminotransferase (AST) at day 2 and day 3 (SI-Fig. 2A,B), and a prolonged elevation of total bilirubin and serum BA (SI-Fig. 2C,D). There was no histologically apparent aggravation of liver damage according to the HE staining (SI-Fig. 2E). Notably, slightly more evident cholangiocyte proliferation was observed in the ligated lobes of the BPL groups.

After day 5 post-operation, the weight increment of the intact lobe became negligible in the PVL group (Fig. 1A), with the FRL remaining constant for the rest of the experiment (SI-Fig. 2F). In light of this, day 5 was chosen as the time for staged resection. Second-stage resection was performed as posterior-caudate-lobe-spearing hepatectomy 5 days after PVL (V-Hx) or BPL (B-Hx). The sham procedure followed by posterior-caudate-lobe-spearing hepatectomy (S-SHx) and the sham procedure followed by whole-caudate-lobe-spearing hepatectomy (S-Hx) were used as controls (SI-Fig. 1-A). At the time of resection, the FLRs were 14.02 ± 0.45% in the B-Hx group, 12.18 ± 0.48% in the V-Hx group, 10.13 ± 0.36% in the S-Hx group, and 4.95 ± 0.26% in the S-SHx group (p > 0.05, SI-Fig. 2F). The survival rates were 0% in the S-SHx group, 26.7% in the S-Hx group, 56.7% in the V-Hx group, and 93.3% in the B-Hx group (p < 0.01; Fig. 1C). In the surviving rats, the liver damage makers and total bilirubin were significantly surpassed in the B-Hx group (SI-Fig. 2G–J), In the S-Hx group, consistent with previous reports^14^,^15^, there was severe vacuolation and patchy necrosis in the remnant liver. The histological damage was significantly mitigated in the V-Hx and B-Hx groups, with the B-Hx group having the least damage (Fig. 1D,E). In sum, BPL accelerated the AHC and significantly improved the safety of staged extended resection. Because all the rats in S-SHx died within 24 hours post-operatively, they were not investigated thereafter.

### BPL induced more intensive intrahepatic bile acid retention than PVL

Post BPL/PVL, the hepatic BA levels changed distinctively. In the atrophied lobes, the hepatic BA levels remained unelevated after PVL, but increased to approximately 2- to 3-fold of the normal level after BPL (Fig. 1F). In the hypertrophied liver, PVL resulted in a peak value of less than 1.5-fold of the normal level, while BPL led to a peak value of 2- to 3-fold (Fig. 1G). Compared with PVL, BPL induced moderate hepatic BA retention in the ligated and the intact liver.

### BPL accelerated hepatocyte mitosis in intact liver involving enhancing activation of FXR, and promoted caspase3-mediated apoptosis in the ligated liver

In the hypertrophied lobes, BPL had significantly higher Ki-67 labelling indexes at days 3 and 5 (Fig. 2A), indicating faster and more sustained hepatocyte proliferation was responsible for the enhanced hypertrophy in the intact lobes. The levels of p21 expression were similar in both groups (Fig. 2B,C), indicating well-tolerated regeneration.

When challenged with BA overload, such as hepatectomy and bile duct ligation, the remnant liver adapts by down-regulating BA synthesis and uptake through FXR-dependent pathways^4^,^16^ to protect liver cells from BA toxicity^16^. In our model, the BA overload after BPL might induce faster and/or stronger activation of the FXR-signalling. The expression of the FXR protein and the transcription of its target genes, including CYP7a1, NTCP, BSEP and OSTβ were investigated. We found that compared with the PVL group, an earlier and stronger up-regulation of FXR expression was observed in the BPL group (Fig. 2B,D), which is accompanied by prompt and sustained repression of transcription of CYP7a1 and NTCP, and more marked and drastic up-regulation of BSEP and OSTβ (Fig. 2E). These results suggested a faster and stronger response of activation the FXR-dependent signalling pathway induced by BPL.

**Figure 3:**
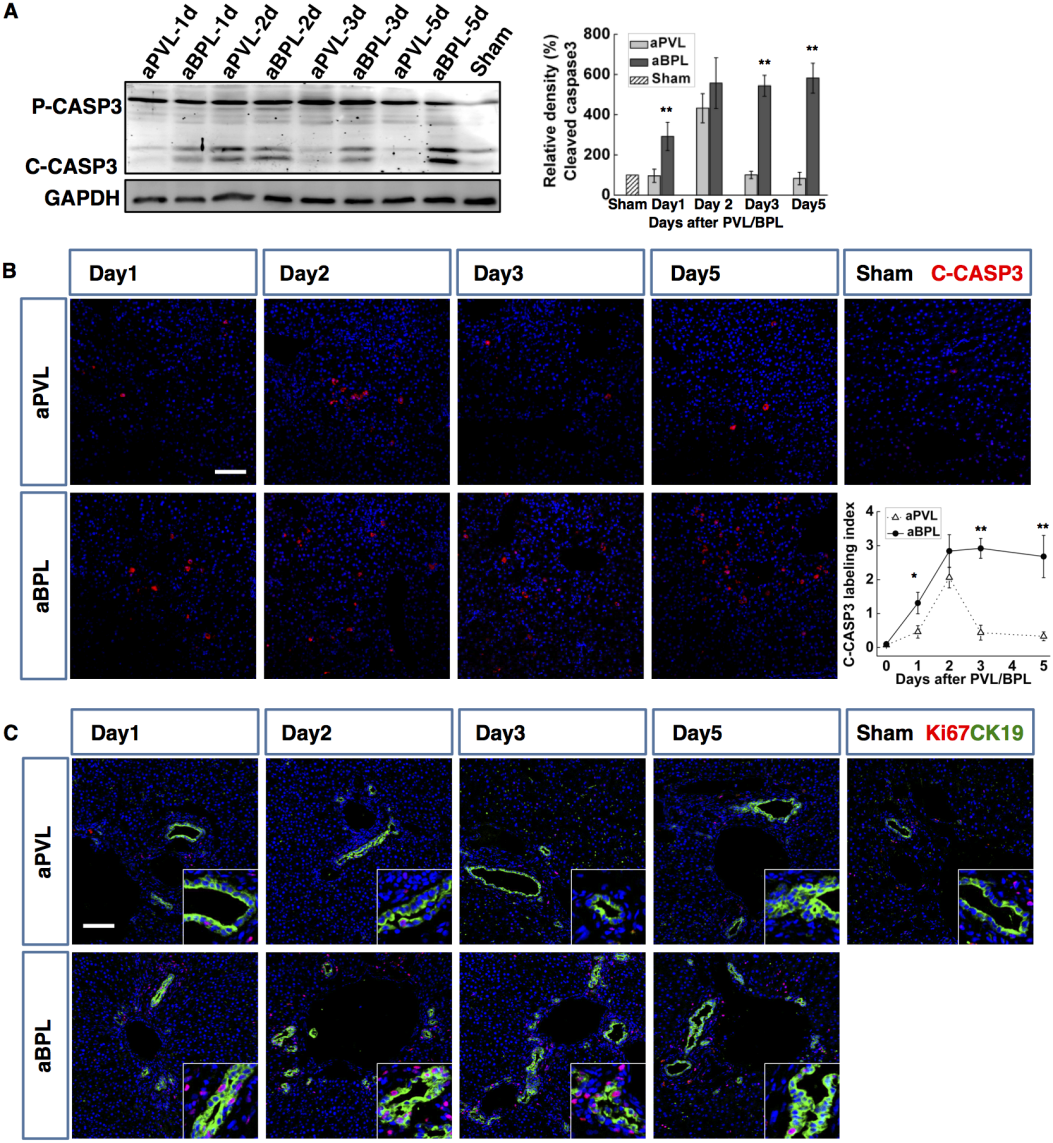
BPL resulted in more intense hepatocyte apoptosis and more evident cholangiocyte proliferation in the atrophied lobes than PVL. Western blotting for caspase-3 in the atrophied lobes after BPL/PVL (indicated as aPVL, aBPL), pro-caspase-3 (Pro-CASP3) and cleaved caspase3 (C-CASP3) were indicated (**A**). Densitometric measurements were acquired from three individual experiments. Immunofluorescence staining of cleaved caspase-3 (C-CASP3) showed single cell apoptosis in both PVL and BPL, the C-CASP3 labelling indexes were calculated (**B**). Double staining of CK19 (green) and Ki67 (red) was performed in the ligated liver (**C**). (Scale bar, 100 μm. The data are presented as the mean ± SD, *p < 0.05, **p < 0.01).

In the atrophied lobes, western blotting for caspase-3 showed a transient cleavage at day 2 after PVL, but prolonged activation in the BPL group (Fig. 3A). Immunofluorescence of cleaved caspase-3 confirmed this observation, revealing scattered single cell apoptosis (Fig. 3B). These suggested that the faster atrophy in the ligated lobes was mediated by apoptosis. The hepatocyte Ki-67 labelling index remained lower than 0.5% in the PVL group but higher than 3% in the BPL group. Consistent with HE staining, more CK19-stained cholangiocytes co-expressed Ki67 in the BPL group (Fig. 3C), indicating more active cholangiocyte proliferation induced by BPL.

### BPL resulted in prompt and stable hepatocyte proliferation after second-stage hepatectomy

**Figure 4:**
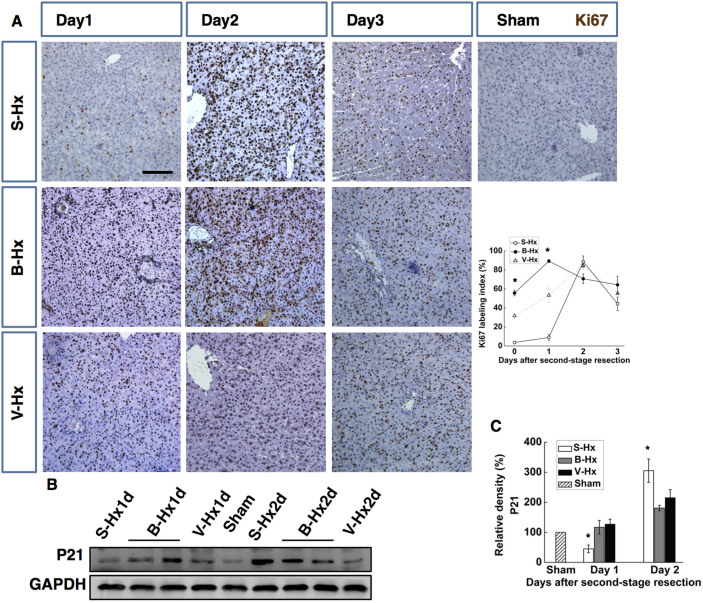
After staged resection, the regenerative response was prompt and efficient in the B-Hx group. Ki67 staining of liver remnant after second-stage resection, with the Ki-67 labelling indexes calculated (**A**). The Ki67 labelling index levels 5 days after sham; BPL, PVL were used as day 0 for S-Hx, B-Hx, and V-Hx, respectively. Western blotting for p21 after second-stage resection (**B**). Densitometric measurements were acquired from six individual experiments (**C**). (Scale bar, 100 μm. The data are presented as the mean ± SD, *p < 0.05, **p < 0.01 Full-length blots presented in SI-Figure 4).

After staged hepatectomy, the S-Hx group was associated with a poor regenerative response, which is consistent with previous reports^14^,^15^,^17^. The Ki-67 labelling index remained lower than 10% at day 1, rose by more than 90% at day 2 and plummeted to less than 50% at day 3. In V-Hx, the Ki-67 labelling index was approximately 30% at the time of resection, raised gradually to higher than 85% at day2 and dropped to 50% at day3, In B-Hx, the Ki-67 labelling index was approximately 60% at the time of resection, raised to higher than 90% at day2, then decreased gradually, to 60% at day3(Fig. 3A). These suggested that a more sustained regenerative response was induced in the B-Hx group, compared with the other groups. The p21-dependent barrier to cell-cycle progression was tested as the main reason for liver failure after extended hepatectomy^18^. Consistently, we found that p21 was extremely suppressed then over-expressed in the S-Hx group, but remained constantly low in the other groups (Fig. 4B,C). These data suggest that inhibited p21 expression contributed to improved regeneration in both V-Hx and B-Hx. In sum, with the largest FLR, the B-Hx group also showed sustained and stable proliferation, providing the most favourable survival rate.

### Bile acid retention was responsible for the enhancement of AHC induced by BPL

**Figure 5:**
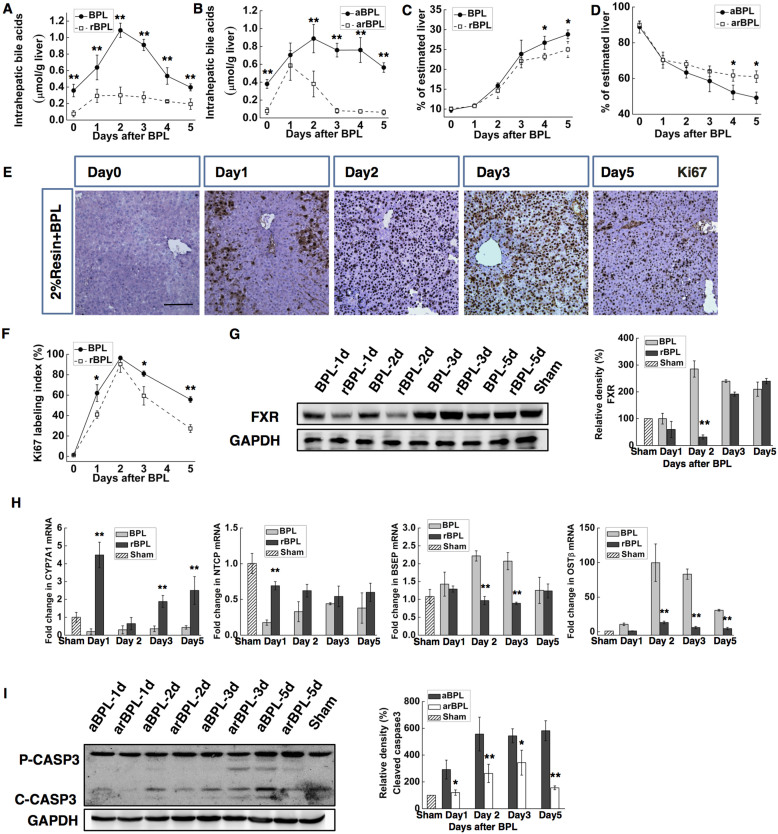
Decreasing the bile acid pool in BPL by 2% resin diet (rBPL) decelerated the AHC. The FXR-dependent acceleration of mitosis was counteracted, and the apoptosis was inhibited. Diet containing 2% resin was fed to rats underwent BPL (rBPL) since 1 day before the ligation till the end of observation, rats underwent BPL with regular diet were used as control. Hepatic bile acid levels in the hypertrophied lobes (**A**), and the atrophied lobes (**B**). Weight of the hypertrophied (**C**) and atrophied (**D**) lobes. Ki67 immunochemical staining of the hypertrophied portion, the Ki-67 labelling indexes were compared with those in the BPL group (**E,F**).Western blotting for FXR revealed delayed activation in the rBPL group (**G**) CYP7a1, NTCP, BSEP and OSTβ mRNA expression levels were measured in the intact liver by quantitative real-time PCR (**H**).Western blotting for caspase-3 in the atrophied lobes (indicated as aBPL and arBPL) revealed decreased apoptosis in the rBPL group, pro-caspase-3 (Pro-CASP3) and cleaved caspase3 (C-CASP3) were indicated (**I**). Densitometric measurements were acquired from 3 individual experiments. (Scale bar, 100 μm. The data are presented as the mean ± SD, *p < 0.05, **p < 0.01 Full-length blots are presented in SI-Figure 4).

**Figure 6:**
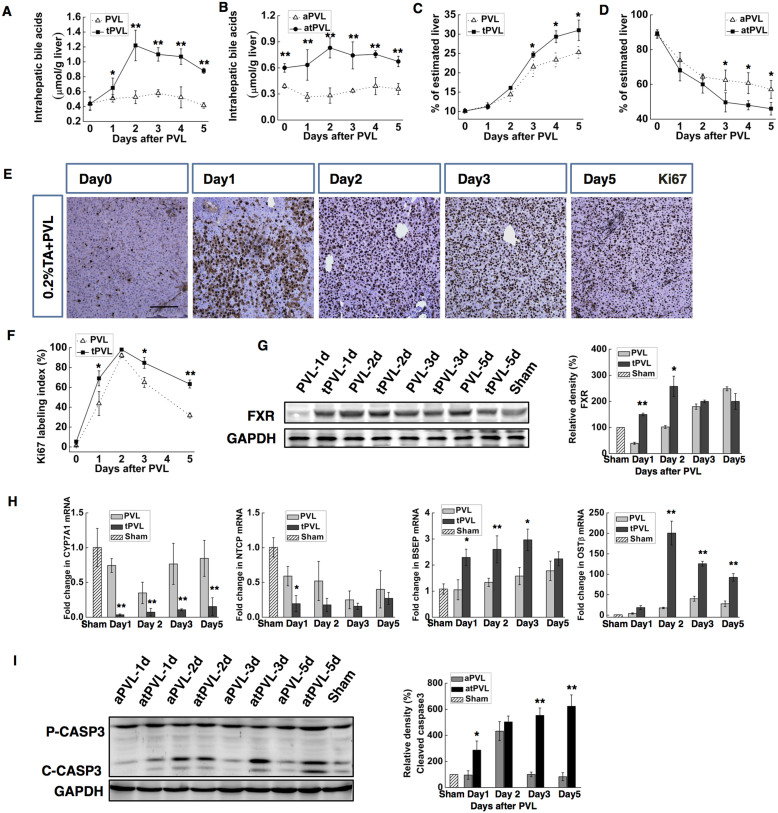
Enhancing hepatic bile acid concentration in PVL by 0.2% taurocholate diet (tPVL) promoted the AHC. The FXR-dependent acceleration of mitosis was induced, and the apoptosis was prolonged and intensified. Diet containing 0.2% taurocholate was fed to rats underwent PVL (tPVL) since 1day before the ligation till the end of observation, rats underwent PVL with regular diet were used as control. Hepatic bile acid levels in the hypertrophied lobes (**A**), and the atrophied lobes (**B**). Weight of the hypertrophied lobes(**C**) and atrophied lobes (**D**). Ki67 immunochemical staining of the hypertrophied portion, the Ki-67 labelling indexes were compared with those in the PVL group (**E,F**).Western blotting for FXR revealed earlier activation in the tPVL group(**G**). CYP7a1, NTCP, BSEP and OSTβ mRNA expression levels were measured in the intact liver by quantitative real-time PCR (**H**).Western blotting for caspase-3 in the atrophied lobes (indicated as aPVL and atPVL) revealed enhanced and prolonged apoptosis in the tPVL group, pro-caspase-3 (Pro-CASP3) and cleaved caspase3 (C-CASP3) were indicated (**I,J**). Densitometric measurements were acquired from 3 individual experiments.(Scale bar, 100 μm. The data are presented as the mean ± SD, *p < 0.05, **p < 0.01. Full-length blots are presented in SI-Figure 4).

The role of BAs in enhancing AHC after BPL was examined more directly in rats in which BA pools were either increased by a 0.2% taurocholate diet or decreased by a diet containing 2% BA sequestering resin cholestyramine. BPL was performed on the second day after diet containing 2% resin (rBPL), and PVL was performed on the second day after diet containing 0.2% taurocholate (tPVL) (SI-Fig. 1C). Compared to BPL, the BA levels were markedly reduced by the administration of resin in the rBPL group, in both serum (SI-Fig. 3A) and the liver parenchyma (Fig. 5A,B). In the rBPL group, the hepatic BA levels remained at normal levels in the intact liver, significantly lower than those in the BPL group at all times investigated. In the ligated liver, the hepatic BA levels were significantly lower in the rBPL group the in the BPL group at day2–5. As a result, the AHC became compromised. At day 4, the weight of the hypertrophied lobes in the rBPL group became significantly lower than in the BPL group, while the atrophied lobes became higher (P < 0.05. Fig. 5C,D). In the hypertrophied lobes, the Ki67 labelling index was slightly lower at day 1 and significantly lower at day 3 and day 5 (Fig. 4E,F). The activation of FXR was also delayed in the rBPL group (Fig. 5G). Consistently, we detected markedly compromised up-regulation of OSTβ and down-regulation of NTCP in the rBPL group. The transcription of BSEP remained constant, while the repressive response of CYP7A1 was replaced by up-regulation with a transient repression at day 2 (Fig. 5H). In the ligated lobes, caspase3 activation also became subtle and transient compared to that in the BPL group (Fig. 4I). On the other hand, taurocholate significantly enhanced BA retention induced by PVL, in both serum (SI-Fig. 3B) and liver parenchyma (Fig. 6A,B). As a result, we detected a remarkable enhancement of the AHC. The differences in the weights of the ligated and the intact lobes between PVL and tPVL were significant at days 3–5(Fig. 6C,D). Compared with PVL, a significantly higher Ki67 labelling index was detected at days 0,1, 3 and 5 in the intact liver (Fig. 6E,F). The expression of the FXR protein in the tPVL group was up-regulated earlier and to a higher level than in the PVL group (Fig. 5G). Compared to the PVL group, we detect extreme repression of CYP7A1 transcription, and more marked down-regulation NTCP in the tPVL group. The up-regulation of OSTβ and BSEP were much more prominent in the tPVL group (Fig. 6H). In the ligated lobes, apoptosis as indicated by caspase 3 cleavage was prominent throughout observation, consistently higher than in the PVL group (Fig. 6I). These results showed that alleviating the BA retention induced by BPL resulted in compromised AHC, while enhancing the BA retention after PVL induced similar effect as BPL, suggesting that the enhanced intrahepatic BA retention was the main reason for the difference between BPL and PVL. Furthermore, we provided direct evidence that increasing the BA levels in the PVL model was sufficient to enhance AHC.

## Discussion

The present study demonstrates that, compared with PVL, BPL significantly promoted the regeneration of the intact liver, and accelerated the atrophy of the ligated liver. Furthermore, BPL resulted in an improved safety profile and more sustained regeneration after second-stage extended hepatectomy, By altering the BA pools in the BPL and PVL models, respectively, we provided direct evidence that BAs were responsible for the observed enhanced AHC.

Consistent with previous reports^2^,^19^, all rats that underwent PVL/BPL survived. Despite a transient elevation of serum liver-damage markers induced by BPL, there was no histological apparent aggravation of liver damage compared to the PVL model. We found no significant weight loss throughout the observation period, in either the PVL group or BPL group, which is consistent with previous reports^2^. Recently, a similar procedure was performed on a patient with liver adenocarcinoma with a satisfactory safety level^20^.This suggested that the procedure could be well-tolerated and readily translatable to clinical practice.

BAs could induce hepatocyte proliferation via FXR signaling^4^,^9^. FXR has been identified as a hepatoprotective receptor that promotes liver repair and regeneration^21^,^22^. In our study, we found that both the BPL and tPVL group showed earlier and more potent up-regulation of FXR, while the PVL and rBPL group showed relatively delayed activation, which are in line with the faster regeneration in the BPL and tPVL groups. In accordance with the affected expression of FXR, the FXR-dependent mechanisms^4^ of BA homeostasis were also altered, including the shut-off of synthesis by CYP7A1 and the induction of export by BSEP^23^. The marked repression of NTCP^24^ and induction of OSTβ^25^,^26^ in the BPL and tPVL groups, which became less prominent in the PVL and rBPL group, further confirmed the involvement of FXR-signalling. Previously, it was reported that elevation of serum BA could predict the effective hypertrophy after portal vein embolisation(PVE) in humans, implicating the involvement of BA signalling^27^. It can be inferred that the imposed BA elevation as induced by BPL would lead to more effective hypertrophy.

Direct cytotoxicity of the BAs is responsible for the liver damage in cholestatic liver disease or after extended hepatectomy^11^,^28^, suggesting that impaired biliary drainage and high BA concentration might be responsible for the enhanced atrophy after BPL. In contrast, we found that hepatic BA levels were similar in the intact and ligated liver, indicating a non-dosage-dependent behaviour. In the tPVL group, the atrophy was also enhanced in the absence of bile duct obstruction, which negated bile duct obstruction as dispensable. We postulated that some portal-delivered substance, e.g., fibroblast growth factor 15(Fgf15)^5^,^22^ might be necessary for the liver to cope with BA overload, without which low BA concentration could enhance hepatocyte apoptosis. Of note, the constant Ki67-stained hepatocytes and cholangiocytes in the atrophied lobes of BPL raised tumour-enhancing concerns^29^,^30^. The influence of BPL on tumour growth is currently under investigation.

Rat 90% hepatectomy is associated with poor regenerative response, high incidence of liver failure and parenchyma necrosis^14^,^15^,^17^,^31^,^32^, and it is used as control in this study (S-Hx). S-Hx is associated with delayed P21 overexpression, which was reported as leading to deficiency in cell-cycle progression; its inhibition could improve survival^18^. In our study, the delayed overexpression of p21 was markedly ameliorated in both V-Hx and B-Hx groups, indicating improved regenerative responses. Notably, the B-Hx group showed the least parenchymal damage according to histology. In addition to high volume of the remnant liver^15^, several other factors should have contributed to such effect. Absence of Fgf15,a BA-induced ileum-derived enterokine, could lead to severe liver necrosis after hepatectomy, while delivery of Fgf15 to the hepatic parenchyma increases survival after extensive liver resection in mice^5^. BA-precondition after BPL might up-regulate the ileum expression of Fgf15 before second-stage resection, thereby reducing parenchyma necrosis after hepatectomy. Fgf15 is also proposed to protect liver from BA overload by switching BA composition toward a more hydrophilic profile^22^. In addition, BA could also activate TGR5 (G-protein-coupled BA receptor1), which protects the BA-overloaded liver through control of BA hydrophobicity^9^. Acute increase in blood BA concentration could stimulate arginine vasopressin (AVP) secretion, which helps to limit portal hypertension and cholestasis^10^. Similarly, the BA-precondition induced by BPL might also activate TGR5 and stimulate AVP secretion, which help to protect the liver parenchyma and improve prognosis.

BPL has been reported as inducing slightly faster AHC than PVL in rats^25^, but it is a more complicated procedure, and it is unknown if its risks outweigh its benefits. Our study demonstrated that BPL could induce faster AHC than PVL through BA-dependent mechanisms, which might contribute to favourable survival after second-stage extended hepatectomy. We therefore propose that BPL may be a safe and efficient future option for patients with insufficient FRL.

## Author Contributions

R.W., G.C. and X.W. designed the study, established the models, analysed and interpreted the data, prepared the figures and wrote the manuscript; K.P. and W.L. performed the experiments, and analysed and interpreted the data; J.D., A.Z. and C.L. conceptualised and designed the study, supervised the project, and revised the paper. All authors vouch for the respective data and analysis, and have approved the final version and agreed to publish the manuscript.

## Supplementary Material

Supplementary Information Supplementary information

## Figures and Tables

**Table 1:** Primer sequences

Genes	Forward(5′-3′)	Reverse(5′-3′)
GAPDH	ACCACAGTCCATGCCATCAC	TCCACCACCCTGTTGCTGTA
FXR	GTGACAAAGAAGCCGCGAAT	GCAGGTGAGCGCGTTGTAAT
BESP	CACTGGGTACATGTGGTGTCTCAT	ATGGCCAATATTCATAGCTGCTAAT
CYP7A1	GCTTTACAGAGTGCTGGCCAA	CTGTCTAGTACCGGCAGGTCATT
OSTβ	TATTCCATCCTGGTTCTGGCAGT	CGTTGTCTTGTGGCTGCTTCTT
NCTP	CGTTGCCGGAATGTTTGTCT	TGCCCTTCTGTCTCAGTTCATG
