# Supplementary material for: Simultaneous bile duct and portal vein ligation induces faster atrophy/hypertrophy complex than portal vein ligation: role of bile acids
Source: Sci Rep. 2015 Feb 13;5:8455. doi: 10.1038/srep08455 (PMC4326731; doi:10.1038/srep08455)
Supplement: Supplementary Information [file srep08455-s1.pdf]

Simultaneous bile duct and portal vein ligation induces faster atrophy/hypertrophy complex than portal vein ligation: role of bile acids

(Laboratory Study)

Weizheng Ren[1#], Geng Chen[2#], Xiaofeng Wang [1 #], Aiqun Zhang[1], Chonghui Li[1], Wenping Lv[1] Ke Pan[1],Jia-hong Dong[1*]

1. Department & Institute of Hepatobiliary Surgery, Chinese PLA General Hospital, Beijing, China.
2. Department of Hepatobiliary Surgery, Xinan Hospital, Third Military Medical University

#These authors contributed equally to this manuscript.

***Corresponding author**: Jia-Hong Dong, MD, PhD, FACS, Department & Institute of Hepatobiliary Surgery, Chinese PLA General Hospital, Beijing 100853, China. E-mail:dongjh301@163.com

Supported by grants from China National Key Technology R&D Program (2012BAI06B01), National Natural Science Foundation (81270526, 81370572) and National Major Project for Infectious Diseases(2012ZX10002-017)

Disclosure: The authors declare no conflicts of interest.

Reprints: Jia-Hong Dong, Hospital & Institute of Hepatobiliary Surgery, Chinese PLA General Hospital, Beijing 100853, China. E-mail:dongjh301@163.com

SI-Figure 1

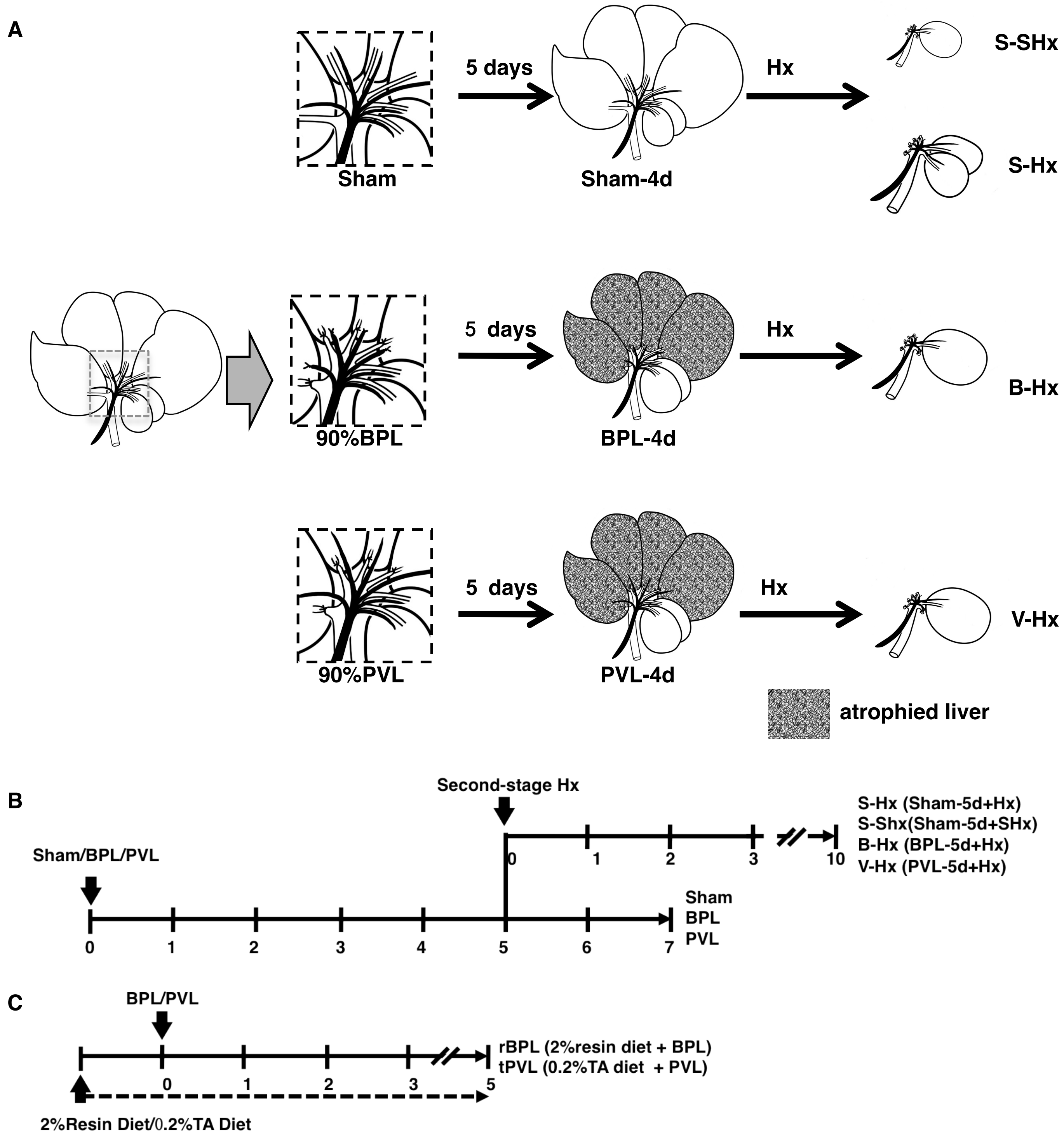

SI-Figure 1 | Surgical Procedures and experimental design. (**A**): Graphical illustration of the surgical operation. Surgical procedures included sham procedure, PVL, in which portal vein of 90% liver were ligated, and BPL, in which bile duct and portal vein of 90% liver were ligated simultaneously. The dotted area is the atrophied liver. Secondary operations preserving the posterior caudate lobe were performed on the fifth day after PVL (P-Hx), BPL (B-Hx), or sham (S-SHx). In an additional group, the S-Hx group, hepatectomy preserving the whole caudate lobe was performed at day5 post Sham. **(B):** Experimental design, rats were randomized into sham, BPL and PVL groups, and sacrificed as indicated. In a separate study, rats underwent sham/BPL/PVL and subsequently hepatectomy, and sacrificed as indicated. In each group, 30 rats were used to determine survival. **(C)** Diets containing 0.2% taurocholate or 2% cholestyramine (resin) were fed to rats underwent PVL (0.2% taurocholate, tPVL) or BPL (2% resin, rBPL), respectively, since 1 day before the procedure till the time they were sacrificed. (The drawings were done by Ren Weizheng)

SI-Figure 2

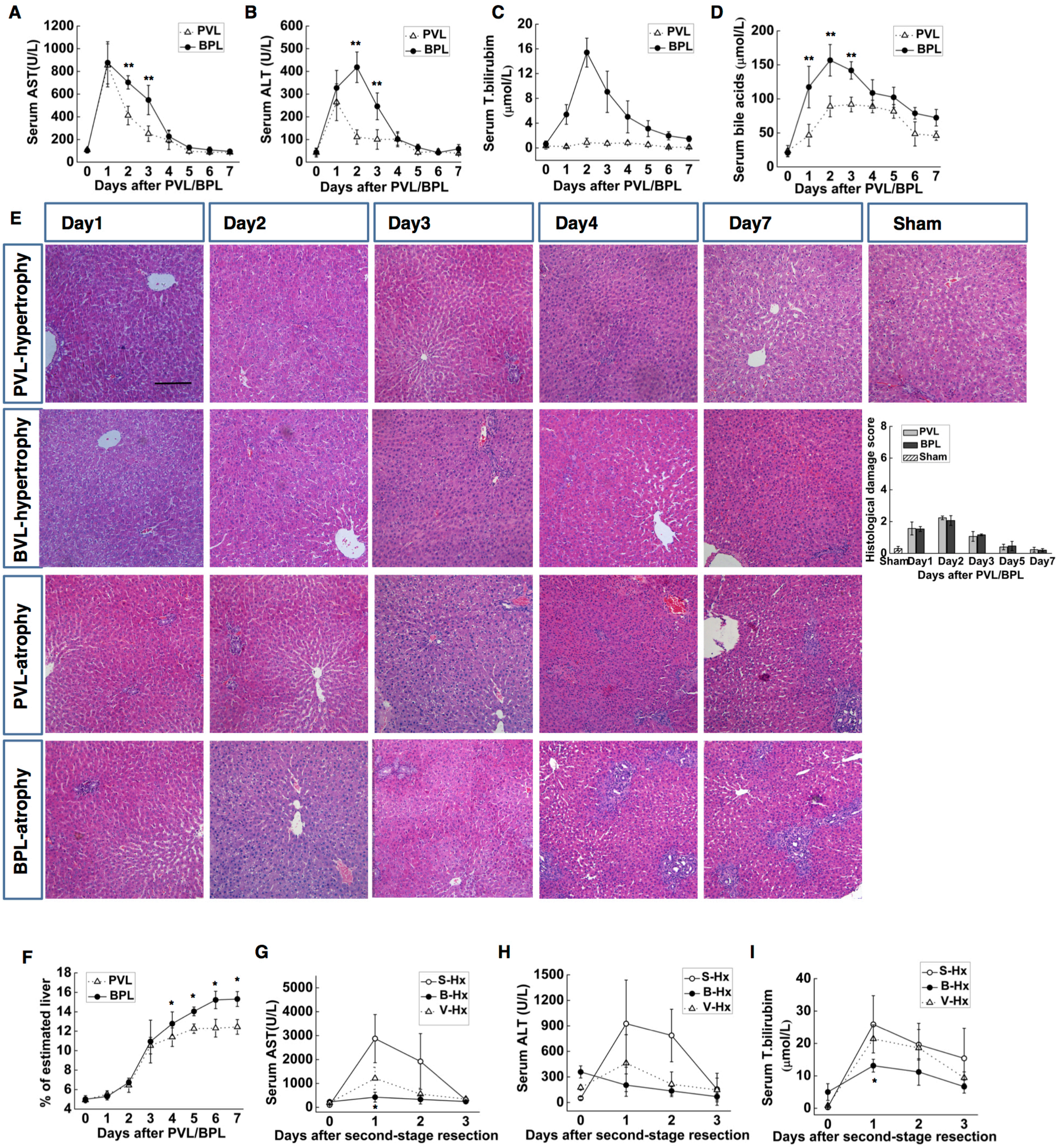

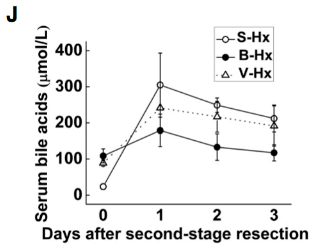

SI-Figure 2 I Despite the transient aggravation of liver damage, prolonged and marked elevation of serum bile acid levels, BPL did not induce evident histological damage compred to PVL. Rats underwent BPL before 90% hepatectomy showed the least elevation of liver damage markers. Serum level of AST**(A)** and ALT**(B)**, tital bilirubin**(C)** and total bile acids **(D)** after PVL/BPL. Histology (HE) of the intact liver was scored as described, and the histology of the ligated liver revealed evident cholangiocyte proliferation in the BPL group**(E)**. Weight of the future liver remnant **(F)** after PVL/BPL. Serum level of AST**(G)** and ALT**(H)**, total bilirubin**(I)**, and total bile acids **(J)** after second stage resection. (Scale bar, 100μm. Data were presented as the mean ± SD, *P<0.05, **P<0.01)

SI-Figure 3

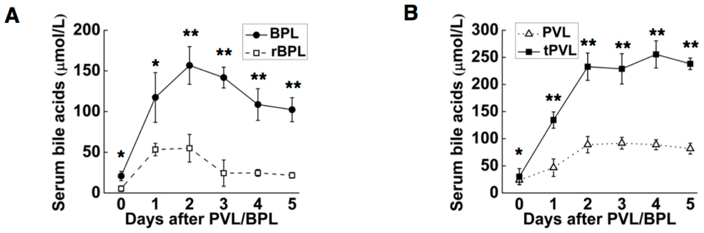

SI-Figure 3 | Serum BA levels in the rBPL and the tPVL groups. Serum BA levels in the rBPL and the tPVL groups were compared with the BPL and PVL group, respectively**(A,B)**.(The data are presented as the mean ± SD, *P<0.05, **P<0.01)

SI-Figure 4

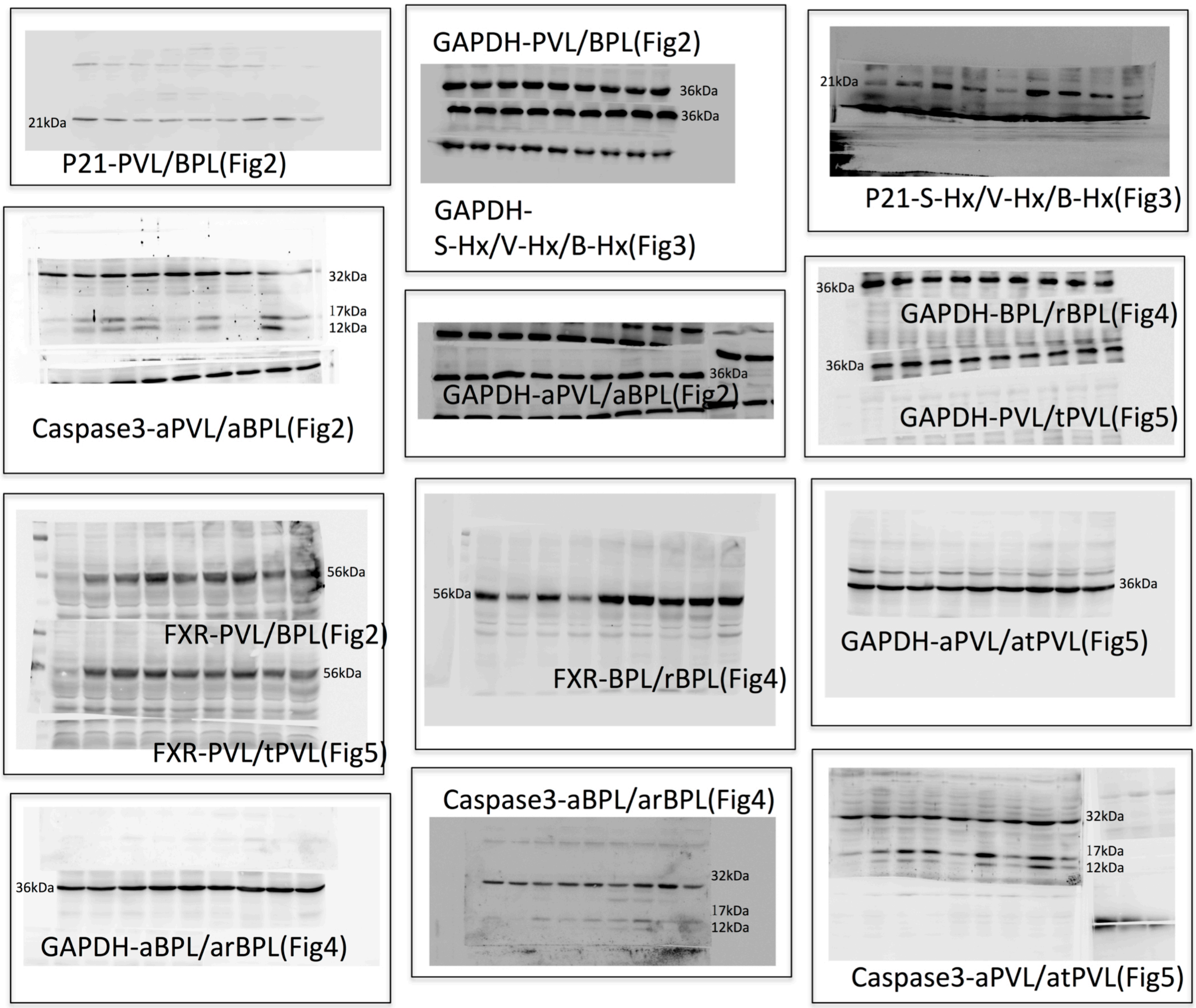

SI-Figure 4 Full-length blots used in the figures with molecular weight indicated.

SUPPLEMENTARY Materials and methods

Animals and Procedures The experiments were performed on 7-week-old male Sprague-Dawley (SD) rats, weighing 220–250g (purchased from the Laboratory Animal Research Center of the Academy of Military Medical Science). The animals were kept in the animal facility of the PLA Medical School which was maintained at 248C with a 12-h light–dark cycle, with access to standardized chow and water ad libitum. Animal procedures were approved by the Institutional Animal Care and Use Committee (IACUC). The rats were fasted overnight for 12 h with free access to water before experiments. All the surgical procedures were performed between 8 AM and 12 AM by an experienced surgeon with sufficient microsurgery training. Ether inhalation was used as anesthesia in all procedures.

Rat liver is lobulated, with the whole caudate lobe accounting for approximately 10% of the total estimated liver weight (3.5% of the net weight). For the analysis of atrophy/hypertrophy complex (AHC) after PVL and BPL, rats are randomized into 2 groups. In BVL group, bile ducts and portal veins of all lobes but the caudate were ligated (90%BPL, **Fig1.A**). In PVL group, only the portal veins of the same portion were ligated(90%PVL, **Fig1.A**). The corresponding portal veins were ligated with 6-0 silk, the bile ducts were double ligated and transected. After the operations, the rats had free access to food and water. Six rats of each group were scarified on postoperative 1-7 day to acquire the growth curve and determine the time for the 2-stage surgery (**Fig1.B**).

The fifth day after PVL/BPL was chosen as the time point for secondary operation, when the difference in weight of the FRL were significant between the two groups. Rats were randomized into 4 groups. The S-Hx group (Sham-90% hepatectomy, S-Hx) underwent initial surgery consisting of manipulation only and followed by hepatectomy preserving the whole caudate lobe 5 days later[1]. In the S-SHx (Sham-subtotal hepatectomy, S-SHx) group, rats underwent sham followed by hepatectomy preserving only the posterior caudate lobe 5 days later. In the V-Hx (PVL before hepatectomy, V-Hx), and B-Hx (BPL before hepatectomy, B-Hx), hepatectomy preserving only the posterior caudate lobe was performed 5 days after 90% PVL or 90%BPL, respectively. Only surviving animals were subjected to analyses. In V-Hx, B-Hx and S-Hx groups, animals were killed under anesthesia at 24, 48 h, and 72h after hepatectomy (n=6). The liver lobes were harvested and then analyzed. . In a separate study, 30 rats were used to examine the survival rate in each of the four groups.

To investigate whether bile acids were responsible for the effect induced by BPL, diets containing taurocholate (0.2%, Sigma Aldrich) or cholestyramine (2%, Sigma Aldrich) were used. Rats switched to diets containing 2% cholestyramine one day before undergoing BPL till sacrificed were adopted to investigate whether enhanced bile acid retention were the mechanism for the effects. Regular diet-fed rats underwent BPL were used as control. Rats switched to diets containing 0.2%taurocholate one day before undergoing PVL till sacrificed were used to reveal whether increasing hepatic bile acids alone were sufficient to induce the effect. Regular diet-fed rats were used as control. (**Fig1.C**).

References

[1] Gaub J, Iversen J. Rat liver regeneration after 90% partial hepatectomy. Hepatology. 1984. 4(5): 902-4.
